# The membrane-associated fraction of cyclase associate protein 1 translocates to the cytosol upon platelet stimulation

**DOI:** 10.1038/s41598-018-29151-w

**Published:** 2018-07-17

**Authors:** Pooja Joshi, David R. J. Riley, Jawad S. Khalil, Huajiang Xiong, Wei Ji, Francisco Rivero

**Affiliations:** 1 School of Life Science and Technology, Changchun University of Science and Technology, Changchun, Jilin China; 2 Centre for Atherothrombosis and Metabolic Disease, Hull York Medical School, Faculty of Health Sciences, University of Hull, Hull, UK; 3 Department of Biochemistry, University of Oxford, Oxford, UK

## Abstract

Platelets undergo profound shape changes upon adhesion to damaged blood vessel walls that are mediated by reorganisation of the actin cytoskeleton in response to receptor-mediated signalling cascades. The highly conserved 56 kDa multidomain cyclase associated protein 1 (CAP1) works in concert with cofilin and profilin to modulate actin filament turnover by facilitating cofilin-mediated actin filament severing and depolymerisation and catalysing profilin-mediated regeneration of actin monomers for reutilisation in growing filaments. CAP1 is abundant in platelets but its roles remain unexplored. We report that in suspended platelets CAP1 localises predominantly at the cell cortex whereas in spread platelets it is uniformly distributed in the cytoplasm, with enrichment at the cell cortex and the periphery of actin nodules. Upon subcellular fractionation most CAP1 was found cytosolic but part associated to the membrane fraction in an actin-independent manner. Interestingly, upon stimulation with thrombin a significant proportion of the membrane-associated CAP1 translocates to the cytosol. This relocalisation was prevented by prior treatment with PGI2 or the nitric oxide donor GSNO, or by inhibition of GSK3. Our results place CAP1 at a crossroad of signalling pathways that control platelet activation by contributing to actin remodelling at the cell cortex and actin nodules during platelet spreading.

## Introduction

Platelets in circulation adopt a discoid shape that is maintained thanks to a highly organised cytoskeleton that includes a peripheral microtubule coil, a network of cross-linked actin filaments that fill the cytoplasmic space and, connected to this, a cortical spectrin-based skeleton associated to the plasma membrane^1^. Platelets undergo profound shape changes when they adhere to the damaged blood vessel wall, first becoming spherical, and then producing filopodia and actin nodules that progress to lamellipodia and stress fibres as the cell spreads and flattens^2^. These changes in platelet morphology are mediated by reorganisation of the actin cytoskeleton in response to multiple receptor-mediated signalling cascades^3^. Platelet activation is accompanied by a rapid increase in the amount of actin assembled into filaments^4^. Numerous proteins with various biochemical activities participate in the dynamics of actin remodelling, including the Arp2/3 complex and their regulators (WAVE, WASP), formins, gelsolin, cofilin, coronin and monomeric actin binding proteins like profilin, β-thymosin and the cyclase associated protein (CAP)^5,6^.

The highly conserved CAP is a 56 kDa multidomain protein that works in concert with cofilin and profilin to modulate actin filament turnover^7^. The C-terminal half harbours two actin-binding domains (a WH2 domain followed by a β-sheet domain) and two proline-rich regions that recruit profilin. This part of CAP competitively displaces cofilin from ADP-actin monomers and catalyses nucleotide exchange by profilin. The N-terminal half enhances cofilin-mediated severing and depolymerisation of actin filaments and is required for hexamerisation^8,9^.

Consistent with that important role in actin turnover, lack of CAP affects cell morphology, polarity, motility and endocytosis, as shown in studies in mammalian cells and various organisms (reviewed in^7^). In mammals two CAP isoforms exist, the ubiquitously expressed CAP1 and the tissue-restricted CAP2, expressed mainly in brain, heart, skeletal muscle, skin and testis^10,11^. CAP1 is a predominantly cytosolic protein, although a small fraction has been found associated to membranes^12^. It usually shows a diffuse cytoplasmic localisation and accumulates in dynamic actin structures at the cell cortex where it co-localises with cofilin^13–15^. Silencing of CAP1 results in accumulation of thick and less dynamic stress fibres, formation of cofilin aggregates and alterations of cell morphology, motility and adhesion, the latter through interaction with talin and focal adhesion kinase (FAK)^15,16^. Additionally mammalian CAP1 has been shown to be a proapoptotic protein^17^. Whilst no mouse knockout model for CAP1 has been described, CAP2 deficient mice show reduced postnatal viability and a series of cardiac and neural morphofunctional defects^18,19^. Primary cells isolated from CAP2 deficient mice display alterations in the formation of protrusions and in actin turnover^20,21^.

Given the central role of actin remodelling for platelet function a deep knowledge of its regulation is warranted. Although CAP1 is an important regulator of the actin turnover and, incidentally, the first mammalian CAP orthologue was first isolated from pig platelet lysates^22^ no reports investigate this protein in platelets. Here we show that CAP1 is an abundant protein in human platelets. CAP1 is mainly cytosolic, but a significant amount associates to membranes in an actin-independent manner and translocates to the cytosol upon stimulation with thrombin. In immunocytochemistry studies CAP1 shows a diffuse cytoplasmic localisation with accumulation at the cell cortex, filopodia and actin nodules. Our study paves the way for further studies towards establishing the role of CAP1 in platelet actin dynamics and platelet function.

## Methods

### Reagents

Primary antibodies against following proteins were used: CAP1 (Abcam ab133655), CD36 (Santa Cruz H-300 sc-9154), Syk (Santa Cruz 4D10 sc-1240), GAPDH (Calbiochem 6C5-CB1001), β-actin (Abcam ab20272), β3-integrin (Santa Cruz HC93 sc-14009), cofilin (Cell Signaling Technology D3F9 #5175), profilin-1 (Cell Signaling Technology #3237), and vinculin (Sigma SAB4200080). Secondary antibodies Alexa Fluor 568- or 488-conjugated anti-mouse and anti-rabbit immunoglobulins (Molecular Probes, Invitrogen Life Technologies Ltd.) were used for immunofluorescence. Peroxidase-conjugated anti-mouse and anti-rabbit immunoglobulins (Sigma-Aldrich Co. Ltd.) or IRDye 680 or IRDye 800 anti-mouse and anti-rabbit immunoglobulins (LI-COR Biosciences, Lincoln, USA) were used for Western blot.

Human fibrinogen was from Enzyme Research (Swansea, UK), collagen (Kollagenreagens Horm) was from Takeda (Osaka, Japan), recombinant human resistin (450-19) was from PeproTech (London, UK), latrunculin B and GSNO were from Enzo Life Sciences (Exeter, UK). PGI2 was from Cayman Chemical (Michigan, USA). Glycogen synthase kinase 3 (GSK3) inhibitor CHIR99021 was from Abcam. Other reagents were from Sigma-Aldrich unless otherwise indicated.

### Platelet preparation

Human blood was taken from drug-free volunteers by clean venepuncture into acid citrate dextrose (ACD) (29.9 mM sodium citrate, 113.8 mM glucose, 72.6 mM NaCl and 2.9 mM citric acid, pH 6.4). Platelet-rich plasma (PRP) was obtained by centrifugation of whole blood at 200 × g for 15 minutes. Platelets were isolated from PRP by centrifugation at 800 × g for 12 minutes in the presence of 6 mM citric acid. Platelets were washed in low pH buffer (0.036 mM citric acid, 0.01 mM EDTA, 0.005 mM glucose, 0.05 mM KCl, 0.09 mM NaCl) and centrifuged at 800 × g for 12 minutes. Sedimented platelets were resuspended in modified Tyrode’s buffer (150 mM NaCl, 5 mM HEPES, 0.55 mM NaH_2_PO_4_, 7 mM NaHCO_3_, 2.7 mM KCl, 0.5 mM MgCl_2_, and 5.6 mM glucose, pH 7.3) and maintained at 37 °C for 45 minutes prior to experiments. The study was approved by the Hull York Medical School Research Ethics Committee and all research was performed in accordance with relevant guidelines and regulations. Informed consent was obtained from all blood donors.

### Platelet fractionation

Washed platelet suspensions (5 × 10^8^ platelets/ml), either untreated or treated with various substances for the appropriate time, were mixed with an equal volume of fractionation buffer (320 mM sucrose, 4 mM HEPES, 0.5 mM Na_3_VO_4_, pH 7.4) supplemented with phosphatase and protease inhibitor cocktail. Latrunculin B (LatB) was used at 20 µM for 20 min to depolymerise F-actin prior to lysis. Samples were subjected to 5 freeze-thaw cycles in liquid nitrogen. Intact platelets were removed by centrifugation at 1,000 × g for 5 minutes at 4 °C before centrifugation at 100,000 × g for 60 minutes at 4 °C. Cytosol and membrane fractions were normalised by volume and analysed by Western blot.

### Detergent insoluble pellet extraction

Washed platelet suspensions (1 × 10^9^ platelets/ml) were lysed in an equal volume of Triton X-100 containing lysis buffer (2% Triton X-100, 10 mM Tris-HCl, 10 mM EGTA) supplemented with protease inhibitors. Lysates were spun at 15,600 × g for 20 minutes (low speed) or 100,000 × g for 1 hour (high speed) to separate the detergent soluble fraction from the detergent insoluble pellet. The fractions were normalised by volume, resolved on 12% SDS-PAGE and analysed by Western blot.

### Western blot

Proteins were resolved by SDS-polyacrylamide gel electrophoresis (PAGE) and blotted onto polyvinylidene difluoride (PVDF) membrane. The membrane was incubated with the relevant primary antibody and either the corresponding peroxidase-conjugated secondary antibody followed by enhanced chemiluminiscence detection (Pierce, Thermo Fisher Scientific Inc.) or the corresponding fluorochrome–labelled secondary antibody and visualised and quantified with a LI-COR Odyssey CLx Imaging System (LI-COR Biosciences, Lincoln, USA).

### Immunostaining and microscopy

Washed platelets in suspension were fixed with an equal volume of ice-cold 4% paraformaldehyde and spun at 3,500 × g for 10 minutes on poly-L-lysine (0.01% in PBS) coated coverslips. Alternatively washed platelets were allowed to adhere and spread for 45 minutes on coverslips pre-coated with fibrinogen (100 µg/ml) overnight and blocked with 0.5% bovine serum albumin (BSA) prior to fixing with 4% paraformaldehyde. Cells were permeabilised with 0.3% Triton® X-100 in PBS for 5 min, and subsequently incubated in PBG (0.5% BSA, 0.05% fish gelatine in PBS) for 30 min at room temperature. Primary and secondary antibodies were diluted in PBG and applied for intervals of 1 h. Cells were washed with PBG several times between each incubation step. F-actin was stained with FITC or TRITC-labelled phalloidin (Sigma-Aldrich Co. Ltd.) or blue CytoPainter (Abcam). The coverslips were mounted on object slides using gelvatol or ProLong Diamond antifade mountant (ThermoFisher Scientific) as embedding media. Coverslips were imaged using a Zeiss ApoTome.2 equipped with an AxioCam 506 and a Zeiss Plan-Apochromat 100×/1.4 objective. Images were processed with Zeiss Zen software.

### Statistical analysis

Data are presented as average ± standard deviation (SD) or standard error of the mean (SEM). Normality was tested and statistics were performed using appropriate non-parametric or parametric tests in Graph Pad Prism V6. P values less than 0.05 were considered significant. The data that support the findings of this study are available from the corresponding author upon reasonable request.

## Results

### CAP1 is abundant in platelets

**Figure 1:**
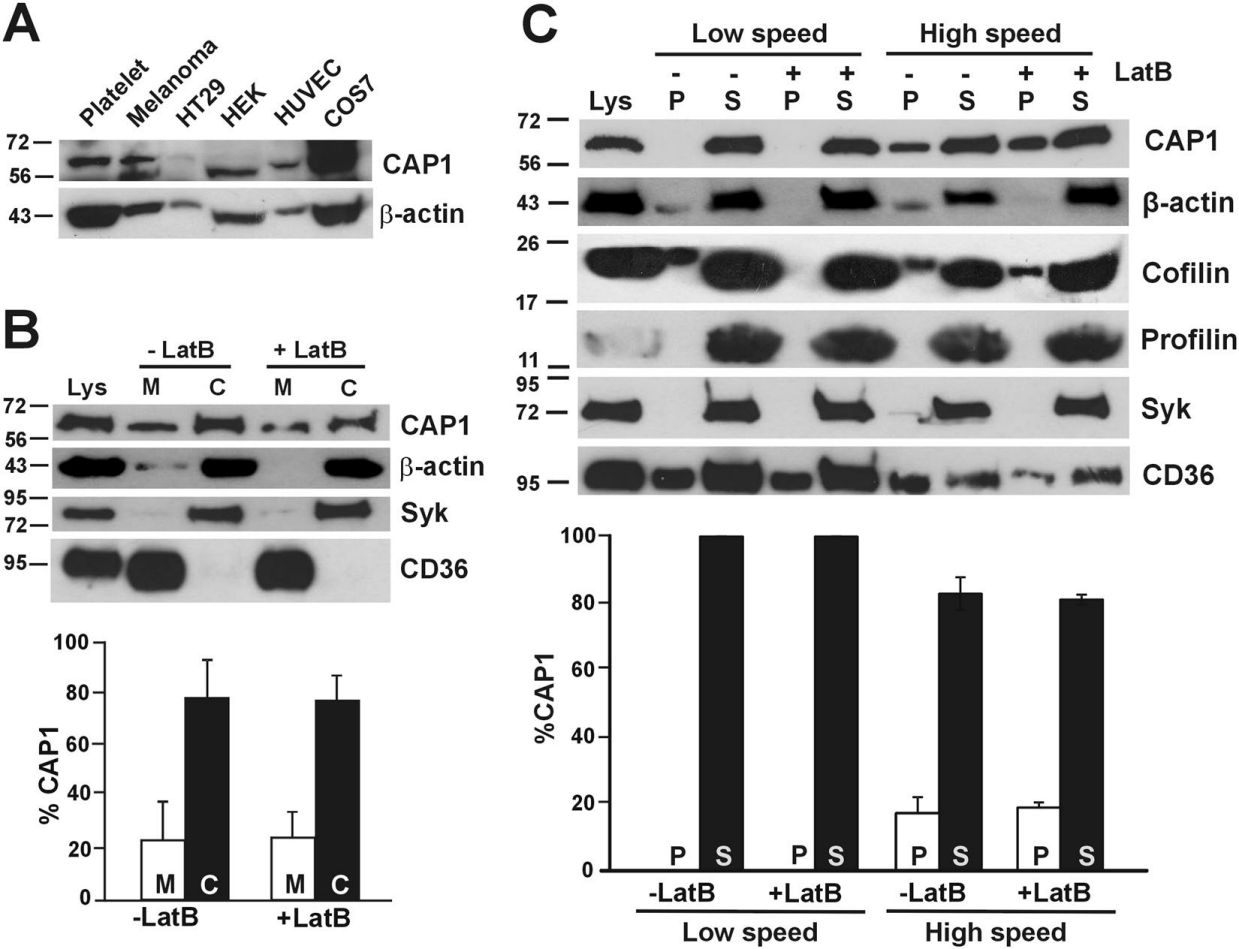
Subcellular distribution of CAP1 in platelets. (**A**) Specificity of anti-CAP1 antibody. Platelet and cell line lysates (30 µg total protein) were resolved by 12% SDS-PAGE, blotted onto PVDF membrane and probed with an antibody for CAP1. The blots were probed for β-actin as a loading control. HT29, human colorectal cancer cell line; HEK, human embryonic kidney 293 cell line; HUVEC, human umbilical vein endothelial cell; COS7, fibroblast-like tissue from monkey kidney tissue. (**B**) Subcellular fractionation. Platelets were lysed by freeze-thaw in liquid nitrogen and spun at 100,000 × g for 1 hour to separate membrane (M) and cytosolic (**C**) fractions. The fractions were normalised by volume and resolved by 12% SDS-PAGE, blotted onto PVDF membrane and probed with antibodies for the indicated proteins. CD36 was used as membrane marker and Syk as a cytosolic marker in resting platelets. Latrunculin B (LatB; 20 µM, 20 min) was used to depolymerise F-actin prior to lysis. CAP1 distribution was quantified by densitometry and expressed as percentage relative to the total (M + C) CAP1 in the lysate. Data represent mean ± SD of three independent experiments. (**C**) Association of CAP1 to actin in detergent insoluble pellet. Platelets (8 × 10^8^/mL platelets) were lysed in the presence of 1% TX-100 and lysates spun at low speed (15,600 × g) for 20 min and high speed (100,000 × g) for 1 hour. Supernatant (S) and pellet (P) were normalised by volume and resolved by 12% SDS-PAGE, blotted onto PVDF membrane and probed with antibodies for the indicated proteins. LatB (20 µM, 20 min) was used to depolymerise F-actin prior to lysis. CAP1 concentration in pellet and supernatant were quantified by densitometry as percent of total (P + S). Data represent mean ± SD of three independent experiments. Full-length blots are presented in Supplementary Fig. 1.

CAP1 is an abundant actin-binding protein ubiquitously expressed in human tissues. To demonstrate the presence of CAP1 in human platelets we resolved platelet lysates along with lysates from various cell lines by SDS-PAGE, blotted and probed with a CAP1-specific polyclonal antibody. CAP1 appeared as a single band with an apparent molecular weight above 56 kDa (Fig. 1A). The antibody we used throughout this study (rabbit monoclonal ab133655) proved specific for human CAP1 but we have demonstrated the presence of CAP1 also in murine platelets using a different antibody (not shown).

To investigate the distribution of CAP1 in platelets we carried out a simple subcellular fractionation. Resting platelets were lysed in an isotonic sucrose solution and cytosol and membrane fractions separated by ultracentrifugation. As shown in Fig. 1B, most of CAP1 (77%) is cytosolic and the rest associates with the membrane fraction. The membrane marker CD36 and the cytosolic marker in resting platelets Syk confirmed that each fraction was free from cross-contamination. Since CAP1 is an actin-binding protein, we further investigated whether this membrane association is mediated by actin. Resting platelets were treated with 20 μM latrunculin B (LatB) to depolymerise F-actin prior to subcellular fractionation. There was no statistically significant difference (Mann-Whitney test) in CAP1 association to the membrane fraction in the absence (22%) or presence (23%) of LatB, indicating that the association of CAP1 to platelet membranes is independent of its association with actin.

To characterise the association of CAP1 to the actin cytoskeleton, resting platelets were lysed in the presence of Triton X-100 and separated into soluble and insoluble fractions by centrifugation at low and high speeds^23^ (Fig. 1C). We observed that CAP1 was not present in the low speed (LS) detergent insoluble pellet, where crosslinked actin filaments and associated proteins sediment, but was recovered in the detergent soluble fraction. At high speed (HS) most CAP1 was soluble, but approximately 17% was found in the detergent insoluble pellet. Notably, no significant difference (Mann-Whitney test) in the amount of CAP1 was observed in the HS pellet when the actin cytoskeleton was depolymerised with LatB prior to centrifugation, indicating that the association of CAP1 to the detergent insoluble pellet is independent of an association to actin.

Because cofilin and profilin function in concert with CAP1, we investigated their behaviour as well. Profilin was recovered in the supernatants at both LS and HS, consistent with its role as monomeric actin binding protein. Cofilin, which interacts with F-actin in addition to G-actin, was observed in HS and LS pellets. It was removed from the LS pellet upon actin depolymerisation, but behaved similarly to CAP1 in the HS pellet. The HS pellet may contain lipid rafts, as suggested by the presence of a fraction of the membrane protein CD36^24^, indicating that the CAP1 and cofilin in the HS pellet are associated to membranes or membrane proteins.

### Localisation of CAP1 in platelets

**Figure 2:**
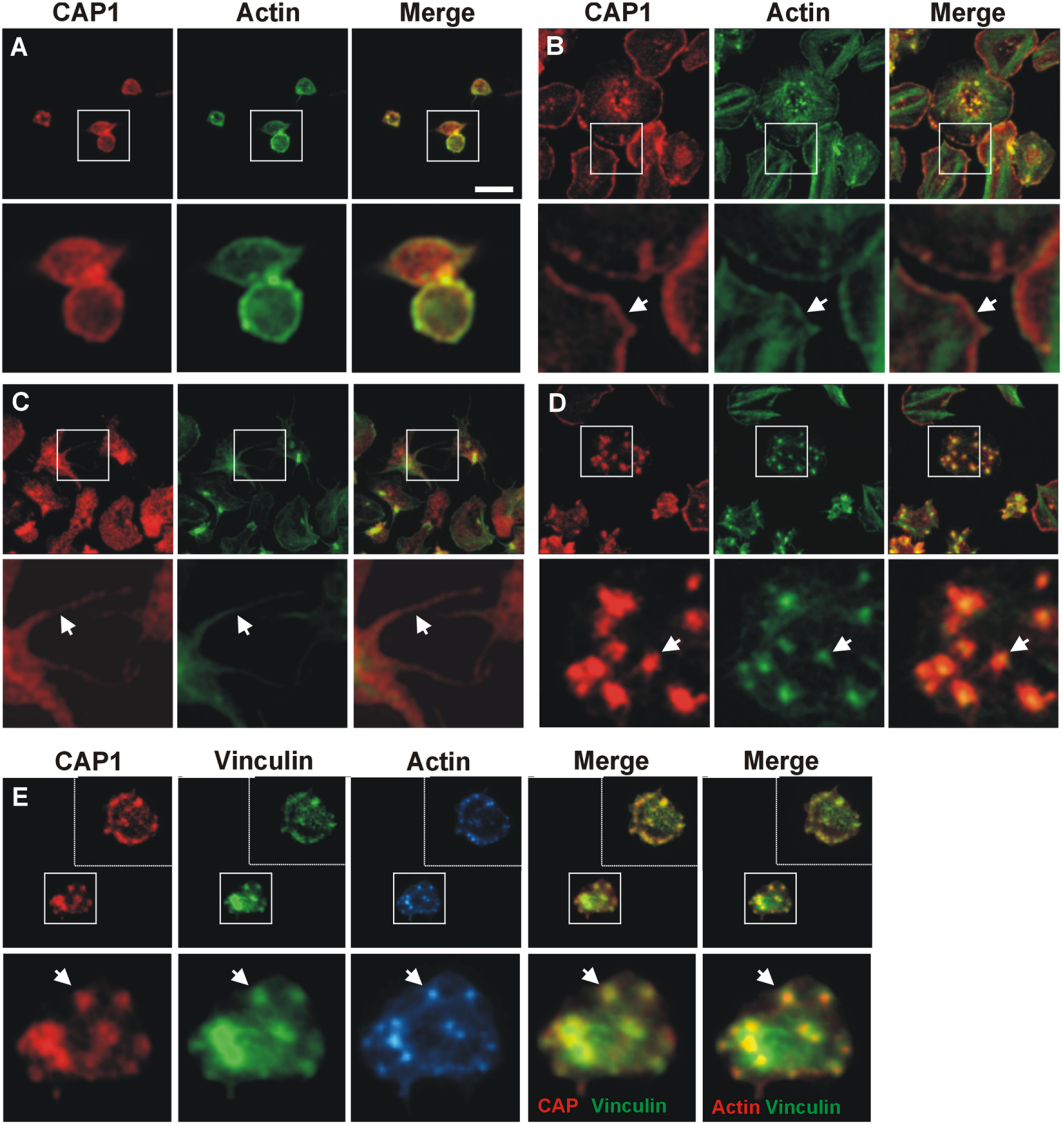
Subcellular localisation of CAP1. Platelets were fixed in suspension with paraformaldehyde and spun on poly-L-lysine coated coverslips (**A**) or were allowed to spread on fibrinogen coated coverslips and fixed with paraformaldehyde (**B**–**E**). For (**A**–**D**) cells were immunostained with an anti-CAP1 antibody followed by an Alexa568-coupled secondary antibody (red) and counterstained with FITC-phalloidin for filamentous actin (green). For E platelets were treated with 100 nM PGI2 5 minutes prior to fixation in order to increase the proportion of cells displaying actin nodules^30^. Platelets were then immunostained with anti-CAP1 and anti-vinculin antibodies followed by Alexa568 or Alexa488-coupled secondary antibodies, respectively (red and green), and counterstained with blue CytoPainter for filamentous actin (blue). Actin colour has been changed to red in the double staining merge panel for better visualisation. Optical sections were acquired with a fluorescence microscope equipped with a structured illumination attachment. Sections were 230 nm apart. Shown is a maximum intensity projection image after deconvolution and single planes of the region are indicated with a square. Arrows point at regions of interest: cell cortex (**B**), filopodia (**C**) and actin nodules (**D**,**E**). Scale bar 5 µm.

We studied the distribution of CAP1 in both suspended and spread platelets by immunostaining and fluorescence microscopy. Resting platelets in suspension have a predominantly discoid shape. In these cells the distribution of CAP1 is predominantly cortical, with a significant proportion of diffuse cytoplasmic staining. CAP1 co-localises with F-actin mainly in cortical regions (Fig. 2A).

In platelets spread on fibrinogen-coated coverslips some CAP1 appears distributed diffusely in the cytoplasm, with conspicuous accumulations in the cortical regions (Fig. 2B), filopodia (Fig. 2C) and actin nodules (Fig. 2D), where it colocalises with F-actin. CAP1-F-actin colocalisation is also observed in central areas, where the platelet granulomere is expected (Fig. 2B). CAP1 is apparently absent from stress fibres. In actin nodules CAP1 displays a broad distribution circling the sharper F-actin staining (Fig. 2D). A co-immunostaing with vinculin, an adhesion protein that localises at actin nodules^25^, showed a clear colocalisation of CAP1 and vinculin at the periphery of the nodules (Fig. 2E).

To visualise the actin-independent association of a fraction of CAP1 to the cell cortex we treated spread cells with the actin-depolymerising drug latrunculin A. This drug caused rounding and retraction of platelets along with fragmentation of stress fibres and weak diffuse phalloidin staining. CAP1 appeared diffusely distributed all over the cell, making the identification of any cortical accumulation impossible (Supplementary Fig. 2).

### CAP1 translocates away from the cell cortex upon platelet stimulation

**Figure 3:**
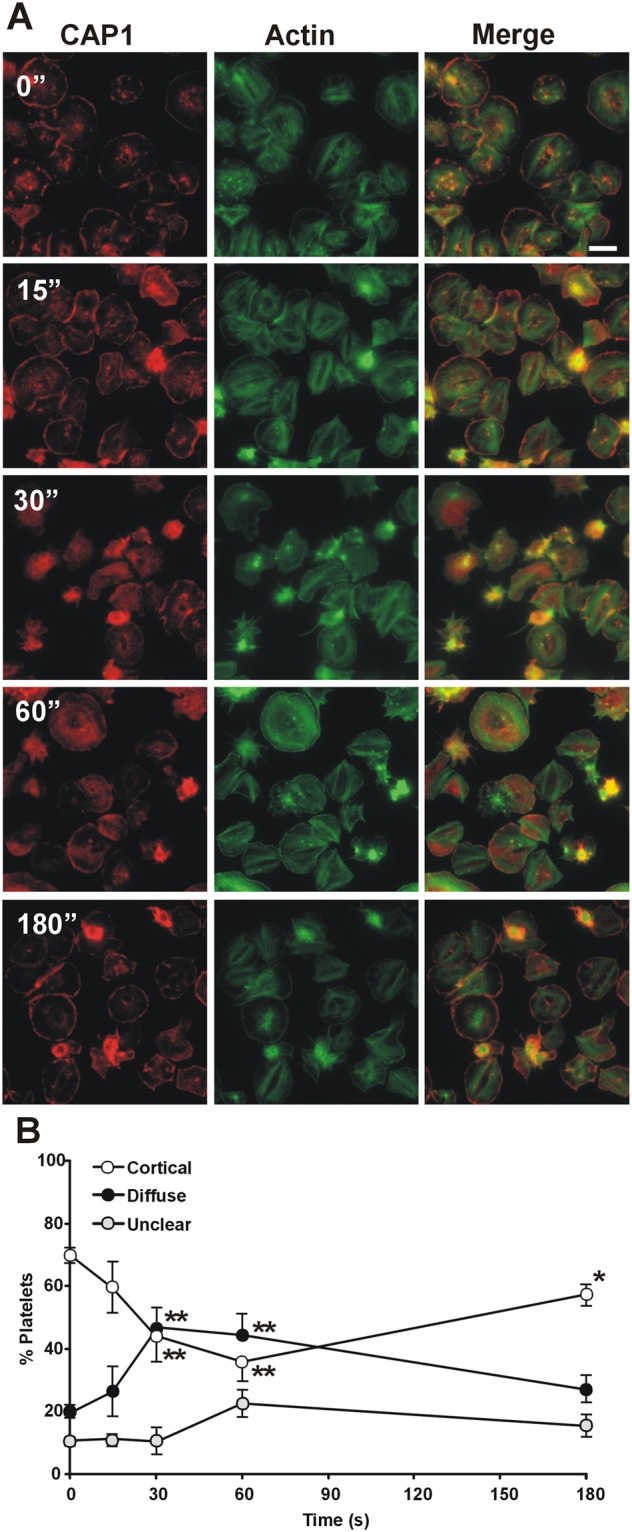
CAP1 relocalises upon thrombin stimulation. (**A**) Platelets were allowed to spread on fibrinogen-coated coverslips and then stimulated with 0.1 U/mL thrombin for the indicated times, fixed with paraformaldehyde and immunostained with an anti-CAP1 antibody followed by an Alexa568-coupled secondary antibody (red) and counterstained with FITC-phalloidin for filamentous actin (green). Images were acquired with a fluorescence microscope equipped with a structured illumination attachment. Scale bar 5 µm. (**B**) Quantification of the pattern of CAP1 distribution upon thrombin stimulation. The proportions of cells with predominantly cortical or diffuse distribution of CAP1 in images like the ones shown in A were calculated from three independent experiments each performed in duplicate coverslips. At least 1000 cells per time point in each experiment were scored. Unclear refers to cells that were not sufficiently enough spread to make a judgment. Data are average ± SEM of four or five independent experiments. *p ≤ 0.05, **p ≤ 0.01, relative to 0 s, Mann-Whitney test.

Thrombin is a potent platelet activator and causes cytoskeletal changes through G-protein coupled receptors PAR1 and PAR4, leading to shape change, aggregation and spreading^26^. To visualise any effect of thrombin stimulation on CAP1 localisation platelets were spread on immobilised fibrinogen prior to stimulation with 0.1 U/mL thrombin for increasing durations before fixation and immunostaining (Fig. 3A). This dose of thrombin was shown to cause the maximum aggregation response in preliminary experiments. In spread platelets CAP1 shows the characteristic subcellular distribution described above (Fig. 2), with 70% cells displaying a predominantly cortical accumulation and 18% showing no particular cortical enrichment (Fig. 3B). Upon thrombin stimulation cortical CAP1 appeared to increasingly disperse with time, so that at 30 seconds the proportion of cells with cortical CAP1 was down to 43%, similar to the proportion of cells with diffuse CAP1 (46%). After 3 minutes CAP1 had partially reverted to the cortex. In these experiments between 11 and 23% of the cells were excluded as they failed to spread, showing a too bright signal to appreciate any clear CAP1 distribution.

In several cell lines CAP1 has been reported to translocate to the outer mitochondrial membrane and promote apoptosis independently of caspase activation^17^. Out attempts to visualise a similar behaviour in platelets were hampered by the small size of these cells in suspension.

**Figure 4:**
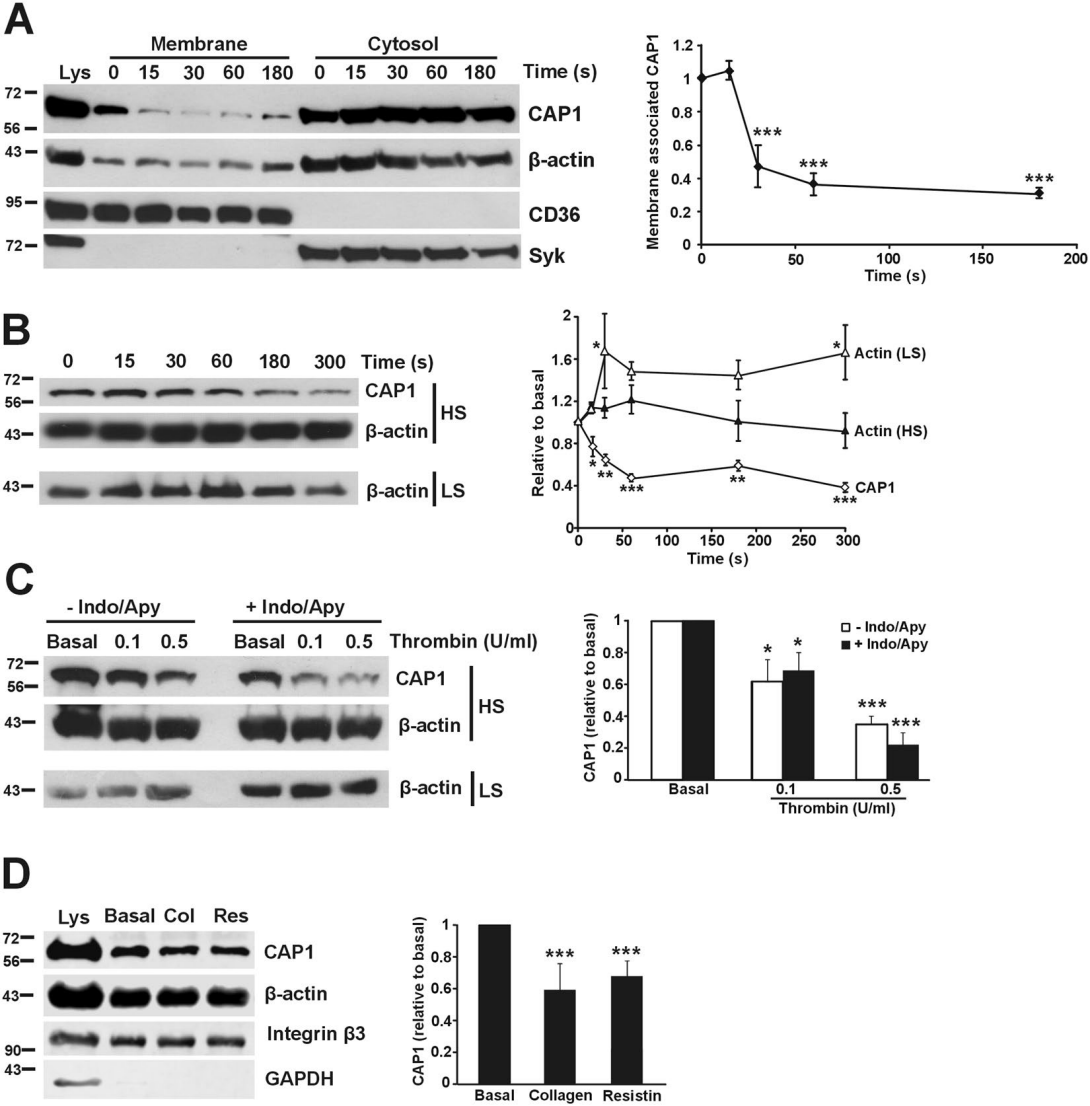
Translocation of CAP1 upon agonist stimulation. (**A**) Reduced membrane-associated CAP1 upon thrombin stimulation. Platelets (8 × 10^8^/mL) were treated with 0.1 U/mL thrombin in the presence of 1 mM EGTA to prevent aggregation for the indicated times prior to lysis and fractionation. Fractions were normalised by volume and resolved on 12% SDS-PAGE, blotted onto PVDF membrane and probed with antibodies for the indicated proteins. CD36, membrane marker; Syk, cytosolic marker. Membrane-associated CAP1 was quantified by densitometry, normalised to CD36 and expressed relative to CAP1 in the 0 s membrane fraction. Data represent mean ± SEM of four independent experiments. (**B**) CAP1 moves away from the high speed (HS) detergent insoluble pellet of thrombin stimulated platelets. Platelets (8 × 10^8^/mL) were stimulated with 0.1 U/mL thrombin prior to lysis in Triton X-100 containing buffer and spun at high speed (100,000 × g) for 1 hour. Only pellets of HS samples were resolved on 12% SDS-PAGE gel, blotted onto PVDF membrane and probed with antibodies for CAP1 and β-actin. CAP1 was quantified by densitometry, normalised to β-actin and expressed relative to the 0 s pellet fraction. The low speed (LS) (15,600 × g) pellet shows the typical increase in actin upon thrombin stimulation. Data represent mean ± SEM of four independent experiments. (**C**) Effect of indomethacin and apyrase on the response of CAP1 in the HS detergent insoluble pellet upon thrombin stimulation. The experiment was performed as in (**B**) in the presence or absence of indomethacin (10 µM) and apyrase (1 U/mL) using the indicated thrombin doses for 1 min. Blots of HS pellets were probed with antibodies for CAP1 and β-actin. The experiment was repeated with centrifugation at low speed (LS) to show the effect of thrombin on actin. CAP1 was quantified by densitometry, normalised to β-actin and expressed relative to the basal pellet fraction. Data represent mean ± SEM of three independent experiments. (**D**) Effect of collagen and resistin on the amount of membrane-associated CAP1. The experiment was performed as in (**A**) using 10 µg/mL collagen for 3 min or 200 ng/mL resistin for 15 min. Only the membrane fractions were analysed. Integrin β3 was used as membrane marker and for normalisation. Data represent mean ± SEM of 4 to 6 independent experiments. For all panels *p ≤ 0.05, **p ≤ 0.01, ***p ≤ 0.001 relative to 0 s or basal. Mann-Whitney test was used for non-parametric data and t-tests were used for parametric data with a Bonferroni correction where appropriate. Full-length blots are presented in Supplementary Fig. 3.

To characterise the behaviour of CAP1 biochemically we stimulated platelets in suspension with 0.1 U/mL thrombin and subjected them to fractionation into membranes and cytosol followed by Western blot analysis (Fig. 4A). A significant reduction in the amount of CAP1 associated to the membrane fraction was observed after 30 seconds of thrombin stimulation. The proportion of membrane-associated CAP1 decreased to as low as 30% of the proportion in resting platelets and persisted for at least 3 minutes. The proportion of actin associated to the membrane fraction did not show apparent changes upon thrombin stimulation, confirming that the behaviour of CAP1 is independent of its association with actin.

We next explored the effect of thrombin in the association of CAP1 to the HS detergent insoluble pellet. Platelets were stimulated with 0.1 U/mL thrombin and the reaction was stopped with lysis buffer at various time points up to 5 minutes. HS pellets were subjected to Western blot analysis (Fig. 4B). We observed a statistically significant time dependent decrease in the proportion of CAP1 in the HS pellet down to 40% of the proportion in resting platelets. The proportion of actin in the HS pellet only showed a very modest increase in the first minute upon thrombin stimulation that did not reach statistical significance. By contrast, as expected, the proportion of actin in the LS pellet rapidly increased upon thrombin stimulation, reached a 1.7 fold peak at 60 seconds and remained elevated afterwards. Again, the behaviour of CAP1 was independent of its association with actin.

To demonstrate that the effects of thrombin on CAP1 re-distribution are primarily due to the agonist and not to secondary mediators we assessed CAP1 localisation in the presence of the thromboxane A2 synthesis inhibitor indomethacin and the ADP degrading enzyme apyrase. Platelets were treated with 10 μM indomethacin and 1 U/ml apyrase for 20 minutes prior to stimulation with two doses of thrombin for one minute and extraction of high speed detergent insoluble pellets (Fig. 4C). We observed a significant dose-dependent translocation of CAP1 from the high speed pellet, with approximately 60% remaining in the pellet with 0.1 U/ml thrombin and 20–35% with 0.5 U/ml thrombin. Both thrombin doses also caused, as expected, an increase in the amount of actin in the low speed pellet. The presence of indomethacin and apyrase did not cause any significant alteration in CAP1 behaviour, indicating that the effect of thrombin is not mediated by secondary mediators.

We next sought to investigate whether the changes in the distribution of CAP1 we saw with thrombin are specific to this agonist. Collagen is one of the key platelet agonists and acts mainly through the GPVI receptor^27^. A collagen concentration of 10 μg/mL, which resulted in maximal aggregation, was used for stimulation followed by subcellular fractionation. We observed that collagen stimulation caused a significant translocation of approximately 40% of CAP1 away from the membrane fraction (Fig. 4D).

Lee *et al*.^12^ proposed that CAP1 is a receptor for resistin, mediating its role in inflammation. Subsequent studies have found an association of resistin and CAP1 with conditions like coronary artery disease and rheumatoid arthritis^28,29^. Sato *et al*.^29^ in particular have shown that resistin contributes to the pathogenesis of rheumatoid arthritis by increasing the production of specific chemokines by fibroblast-like synoviocytes in a CAP1 dependent manner. We have observed that resistin pre-treatment causes an attenuation of platelet aggregation in response to low doses of thrombin (manuscript in preparation). These observations prompted us to investigate the effects of resistin treatment on CAP1 distribution. Platelets were treated with 200 ng/mL resistin for 30 minutes, which we have determined to cause the maximum inhibition of platelet aggregation, followed by subcellular fractionation (Fig. 4D). Upon those conditions we noticed a significant translocation of approximately 35% of CAP1 away from the membrane fraction, suggesting that some of the effects of resistin on platelets may be mediated by CAP1.

### Inhibitory signalling pathways and GSK3 inhibition prevent CAP1 translocation

**Figure 5:**
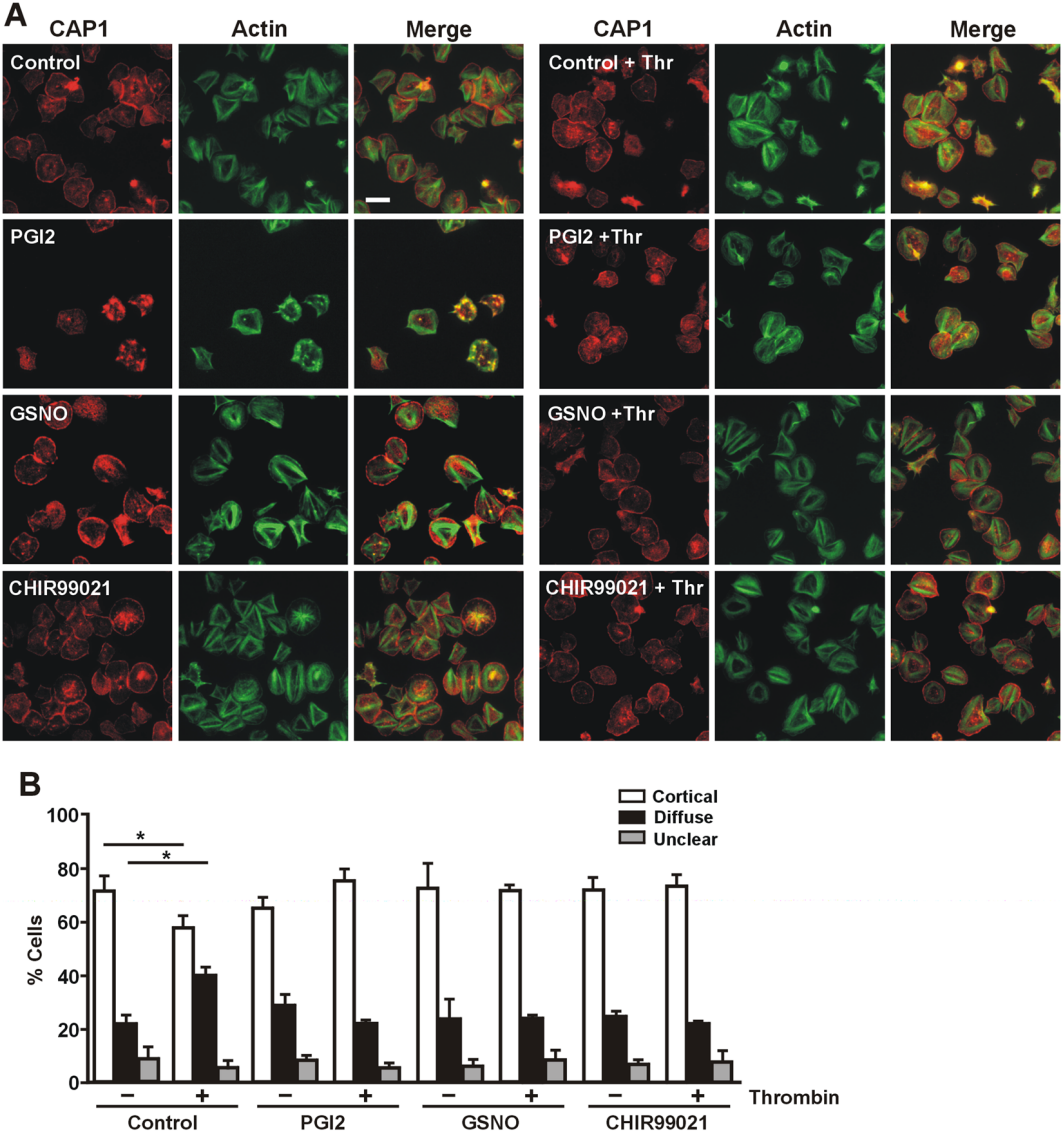
Effects of PGI2, GSNO and GSK3 inhibition on CAP1 relocalisation upon thrombin stimulation. Platelets were allowed to spread on fibrinogen-coated coverslips and then treated with 100 nM PGI2 for 5 minutes, 10 µM GSNO for 20 minutes or 2 µM CHIR99021 for 10 minutes prior to stimulation with 0.1 U/mL thrombin for 30 seconds. Platelets were fixed with paraformaldehyde and immunostained with an anti-CAP1 antibody followed by an Alexa568-coupled secondary antibody (red) and counterstained with FITC-phalloidin for filamentous actin (green). Images were acquired with a fluorescence microscope equipped with a structured illumination attachment. Scale bar 5 µm. (**B**) Quantification of the pattern of CAP1 distribution before (−) and after (+) thrombin stimulation. The proportions of cells with predominantly cortical or diffuse distribution of CAP1 in images like the ones shown in A were calculated from 2 to 5 independent experiments each performed in duplicate coverslips. At least 1000 cells per condition in each experiment were scored. Unclear refers to cells that were not sufficiently enough spread to make a judgment. Data are average ± SEM. *p ≤ 0.05 relative to the respective population not stimulated with thrombin, Mann Whitney test.

Platelets in circulation are continually subject to the effects of inhibitory agents produced by the endothelial cells, namely prostacyclin (PGI2) and nitric oxide, which provoke increases in cAMP and cGMP respectively, and prevent spontaneous platelet activation. We speculated that increased cyclic nucleotide levels would prevent the thrombin-stimulated translocation of CAP1. To test this hypothesis we treated spread platelets with 100 nM PGI2 for 5 minutes or 10 µM S-nitrosoglutathione (GSNO), a nitric oxide donor, for 20 minutes prior to stimulation with 0.1 U/mL thrombin for 30 seconds (Fig. 5A). PGI2 caused a reduction of the proportion of cells with stress fibres and increased abundance of actin nodules, and this effect was less conspicuous with GSNO, as previously described^30,31^. Neither PGI2 nor GSNO per se altered the proportion of cells with predominantly cortical CAP1 distribution (about 70%) (Fig. 5B), however both prevented the effect of thrombin stimulation, which in control cells caused a reduction of the proportion of cells with cortical CAP1 to 56% and a concomitant almost two-fold increase in the proportion of cells with absent cortical CAP1 from 21% to 39%.

Zhou *et al*.^32^ have uncovered a phosphorylation-dependent cycle mediated by GSK3 and other kinases that regulates CAP1 association with cofilin and actin and, notably, also its subcellular localisation. More specifically, in pancreatic cells inhibition of GSK prevented CAP1 enrichment at leading edges and cortical areas. Platelets express predominantly the beta isoform of GSK3, which is tonically active in resting cells^33^, and external stimuli like thrombin cause phosphorylation and inhibition of this kinase. To investigate whether the effect of GSK3 on CAP1 localisation is conserved in platelets we first treated spread platelets with the GSK3 inhibitor CHIR99021 (2 µM) for up to 10 minutes and scored the proportions of cells with predominantly cortical or diffuse CAP1 localisation at various time points. These experiments did not reveal any significant deviation from the results obtained in control platelets (Supplementary Fig. 4). We next investigated whether inhibition of GSK3 would have an effect on thrombin-stimulated translocation of CAP1. We treated spread platelets with 2 µM CHIR99021 for 10 minutes prior to stimulation with 0.1 U/mL thrombin for 30 seconds (Fig. 5A) and observed that GSK3 inhibition prevented CAP1 translocation upon thrombin stimulation (Fig. 5B). Our results suggest that GSK activity has little influence on CAP1 localisation in resting platelets, but is required for CAP1 translocation upon agonist stimulation.

## Discussion

We present immunological evidence of the presence of CAP1 in human platelets, where it appears to be abundant. Proteomics studies place CAP1 among the top 100 most abundant proteins in platelets^34,35^. Burkhart *et al*.^35^ estimate the abundance of CAP1 in 41,700 copies per platelet, approximately one copy per 600 actin monomers. This indicates that CAP1 is unlikely to be an important actin monomer sequestering protein in platelets *in vivo*, an activity that would require equimolar concentrations of CAP1 and G-actin. In sub-stoichiometric amounts relative to G-actin like the ones in platelets, CAP1 would be expected to accelerate the addition of actin monomers to barbed ends^14^. Based on proteomics and transcriptomics studies CAP2 does not appear to be expressed in human platelets and is expressed at very low levels in murine platelets, where CAP1 is also abundant^35–37^.

Although CAP is usually described as a cytosolic protein, in platelets approximately 25% associates with membranes and this association is independent of the actin cytoskeleton. In support of our findings, proteomics studies of enriched platelet plasma membranes clearly identify CAP1^38,39^. Notably, these studies also identify cofilin and profilin in the plasma membrane proteome. Interestingly, the study of Moebius *et al*.^38^ identified CAP1 after applying a purification protocol that significantly reduced the amount of cytoskeleton proteins prior to analysis. This is in agreement with our data showing that CAP1 remains in the membrane fraction after disassembly of the actin cytoskeleton by LatB. An association of a small proportion of CAP1 with the membrane fraction has been reported in the human monocytic leukemia cell line THP-1 and is likely to represent a common feature of CAP^12^.

As expected for a G-actin binding protein, CAP1 does not fractionate in the LS detergent insoluble pellet. A significant proportion does however fractionate in the HS pellet. Again, this association was not disrupted by LatB, indicating that it is independent of actin. The fraction in the HS pellet may correspond to CAP1 associated to membrane-containing structures, probably lipid rafts. Consistent with this, CAP1 has been identified in lipid rafts extracted from mouse brain^40^.

CAP1 displays a conspicuous enrichment at the cortex of both suspended and spread platelets, where it co-localises with F-actin, although this enrichment is more apparent in spread platelets. Similarly, in several mammalian cell lines, as well as in the social amoeba *Dictyostelium discoideum*, CAP1 displays predominantly a diffuse cytoplasmic localisation, but it also accumulates at actin-rich membrane ruffles and lamellipodia^13–16,32,41^. We did not detect CAP1 in stress fibres, in agreement with reports of a small fraction of CAP1 in stress fibres in a few cell lines only, suggesting that an interaction of CAP1 with F-actin is most likely transitory, possibly dependent on its association to cofilin^13–15^. CAP1 localisation in central areas of the spread platelet suggests a role for this protein in vesicle trafficking events associated with degranulation, a process that requires F-actin disassembly^42^.

We report for the first time an accumulation of CAP1 in actin nodules, podosome-related structures visible during early adhesion and spreading^2^. They consist of an actin and Arp2/3 complex core devoid of integrins and surrounded by a ring rich in focal adhesion molecules like talin and vinculin^25^. Interestingly, in HeLa cells CAP1 co-immunoprecipitates focal adhesion kinase (FAK) and talin and appears to interact directly with talin^16^. We observe an accumulation of CAP1 in the ring structure, where we speculate it would be recruited by an interaction with talin. We propose that CAP1 may contribute to the actin turnover of these highly dynamic structures through its actin filament depolymerising and monomer regeneration activities.

Intrigued by the association of CAP1 to the platelet membrane fraction (most likely corresponding to the plasma membrane, as we were unable to distinguish any immunolocalisation pattern that suggests a particular organelle) we sought to visualise changes in CAP1 distribution upon stimulation with the strong agonist thrombin. We observed a fast translocation of CAP1 away from the cell cortex of spread platelets that is mirrored by a depletion of the protein from the membrane fraction and from the HS pellet in platelets in suspension. As we have determined that the HS pellet contains CAP1 in an actin-independent manner, the translocated CAP1 must correspond to membrane-associated CAP1. Moreover, CAP1 translocation parallels the course of actin polymerisation, suggesting that both events are linked to common signalling pathways. We have no explanation to the fact that CAP1 seems to revert to the cortex of spread platelets three minutes after stimulation while translocation persists in platelets in suspension.

Collagen stimulation also provoked CAP1 translocation from the membrane to the cytosol fraction, suggesting that this phenomenon is triggered by a common signalling step downstream of the respective receptors for thrombin and collagen. Similarly, signals downstream of both cAMP and cGMP prevent CAP1 translocation. It is well known that the respective cyclic nucleotide dependent kinases PKA and PKG both share numerous substrates and prevent platelet activation by diverse agonists^43^. A fraction of CAP1 may be associated to one or more membrane receptors or associated proteins but at this moment we ignore the functional relevance of this putative association. Lee *et al*.^12^ have proposed recently that CAP1 is a receptor for the adipokine resistin in monocytic cells, where it mediates the inflammatory response by promoting an increase of cAMP levels. While it is debatable how circulating resistin may interact directly with CAP1, which is not exposed to the cell outside, we observed that exposure of platelets to resistin provoked a comparable translocation of CAP1 to the cytosol. This is in contrast with the effect of resistin reported on a leukemia cell line^12^. Resistin may have effects on platelets different from the ones it elicits in other cell lines that would be worth investigating.

Quinn *et al*.^44^ have recently reported that CAP1 co-immunoprecipitates several adenylyl cyclase (AC) isoforms (at least AC1, 3, 4 and 7) in pancreatic cancer cells and forms an AC3/CAP1/G-actin complex upon stimulation with forskolin that inhibits cell motility. Human platelets express mainly the transmembrane AC6, but also AC3 and AC5 and possibly AC7 and AC9 based on transcriptomics and proteomics studies^35,36^. We were unable to immunoprecipitate AC6 from platelet lysates due to unsatisfactory antibody performance and possibly also low abundance (estimated in 2500 copies per platelet). Should an interaction of CAP1 with ACs in platelets reproduce, we hypothesise that a fraction of CAP1 may be associated to these enzymes at the plasma membrane, contributing to their action and therefore to their inhibitory effects. Stimulation with agonists like thrombin and collagen would disrupt that interaction, shifting the balance towards platelet activation.

The mechanisms controlling CAP1 localisation and translocation are poorly understood. Phosphorylation appears to play a role in pancreatic cancer cells, where CAP1 is a substrate of GSK3 and inhibition of this enzyme resulted in abolished enrichment at leading edges^32^. We did not observe a similar effect in platelets treated with CHIR99021, however inhibition of GSK3 prevented CAP1 translocation to the cytosol in response to thrombin, indicating that although GSK activity is not required basally, it is upon thrombin stimulation. The exact mechanism requires further clarification, as thrombin activation has been shown to inhibit GSK3 in platelets^33^. In fact phosphorylation by GSK3 appears not to be the only regulator of CAP1 translocation because a non-phosphorylatable CAP1 mutant (S307A/S309A) also shows lack of cortical enrichment upon GSK3 inhibition in pancreatic cancer cells^32^.

In summary, we provide evidence that CAP1 is an abundant cytoskeleton regulator in platelets, where it may play roles at the cell cortex and actin nodules during platelet spreading. A fraction of CAP1 remains associated to the membrane in an actin-independent manner and translocates to the cytosol in response to agonists, placing it at a crossroad of the signalling pathways that control platelet activation. Verification of those roles in platelets will require studies in an appropriate animal model lacking CAP1.

## Electronic supplementary material

Supplementary Figures
