# Supplementary material for: The membrane-associated fraction of cyclase associate protein 1 translocates to the cytosol upon platelet stimulation
Source: Sci Rep. 2018 Jul 17;8:10804. doi: 10.1038/s41598-018-29151-w (PMC6050311; doi:10.1038/s41598-018-29151-w)
Supplement: Supplementary file 1 — Supplementary Figures [file 41598_2018_29151_MOESM1_ESM.pdf]

Supplementary Figures

The membrane-associated fraction of cyclase associate protein 1 translocates to the cytosol upon platelet stimulation

Pooja Joshi, David Riley, Jawad S. Khalil, Huajiang Xiong, Wei Ji and Francisco Rivero

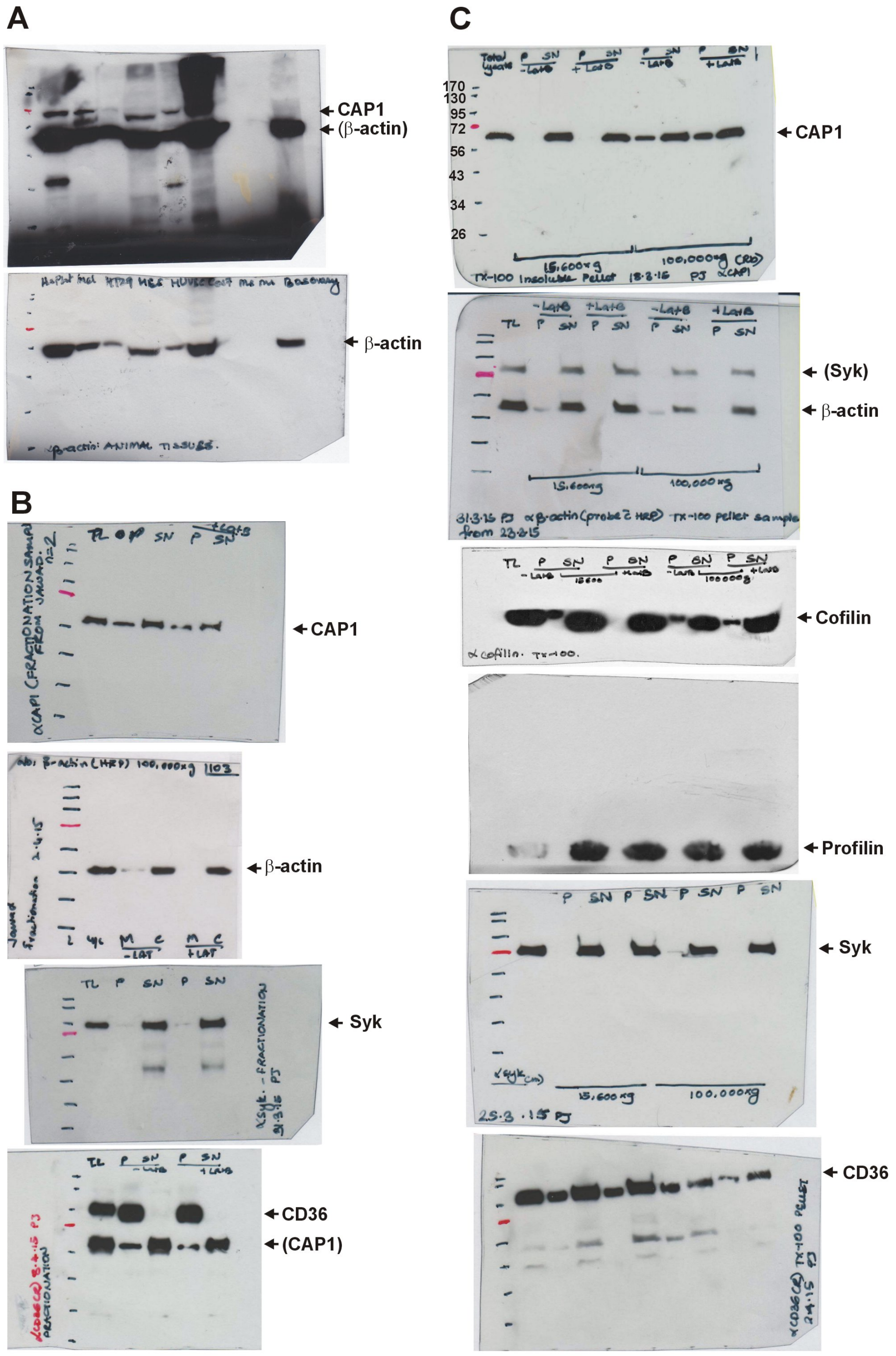

Supplementary Figure 1. Full-length blots corresponding to Figure 1. The blots have been arranged in the same order as they appear in the figure. Note that some blots have been re-probed to visualise a second protein, in which case the first protein (in brackets) may appear over or underexposed. The first blot of panel C displays the band sizes of the protein ladder as an example. For cofilin the lower third of the membrane was cut and used for probing.

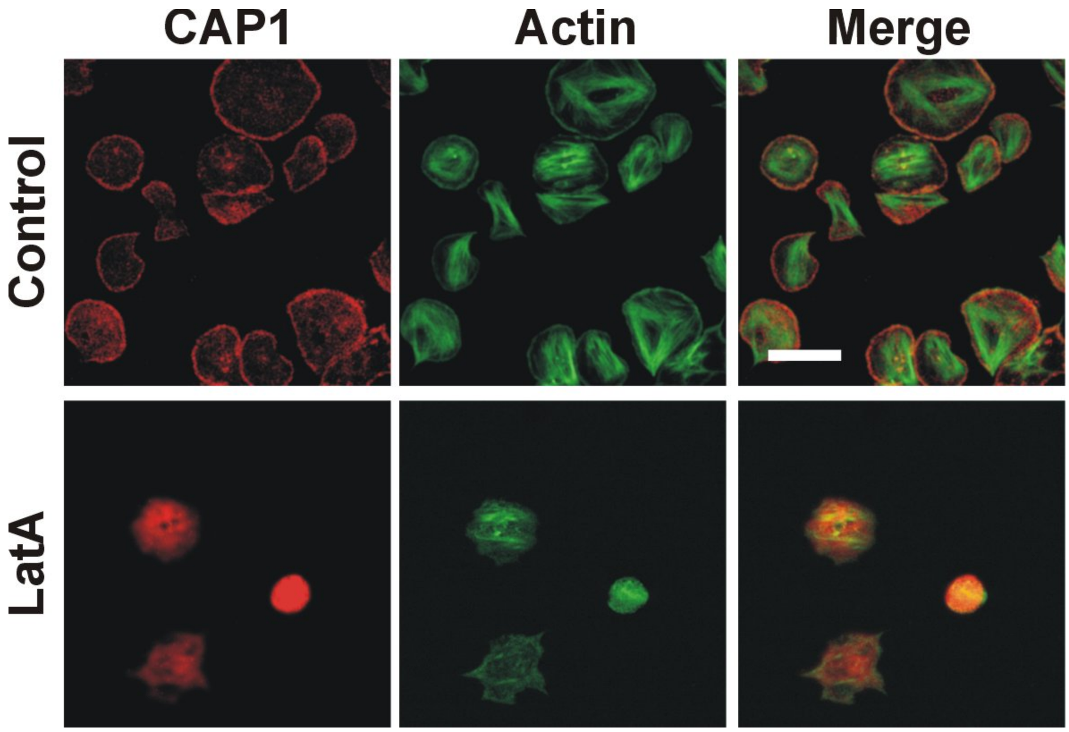

Supplementary Figure 2. Effect of latrunculin A on CAP1 distribution. Platelets were allowed to spread on fibrinogen-coated coverslips and then treated with 3 µM latrunculin A for 30 minutes. Platelets were fixed with paraformaldehyde and immunostained with an anti-CAP1 antibody followed by an Alexa568-coupled secondary antibody (red) and counterstained with CytoPainter for filamentous actin (colour changed to green). Images were acquired with a fluorescence microscope equipped with a structured illumination attachment. Scale bar 10 µm.

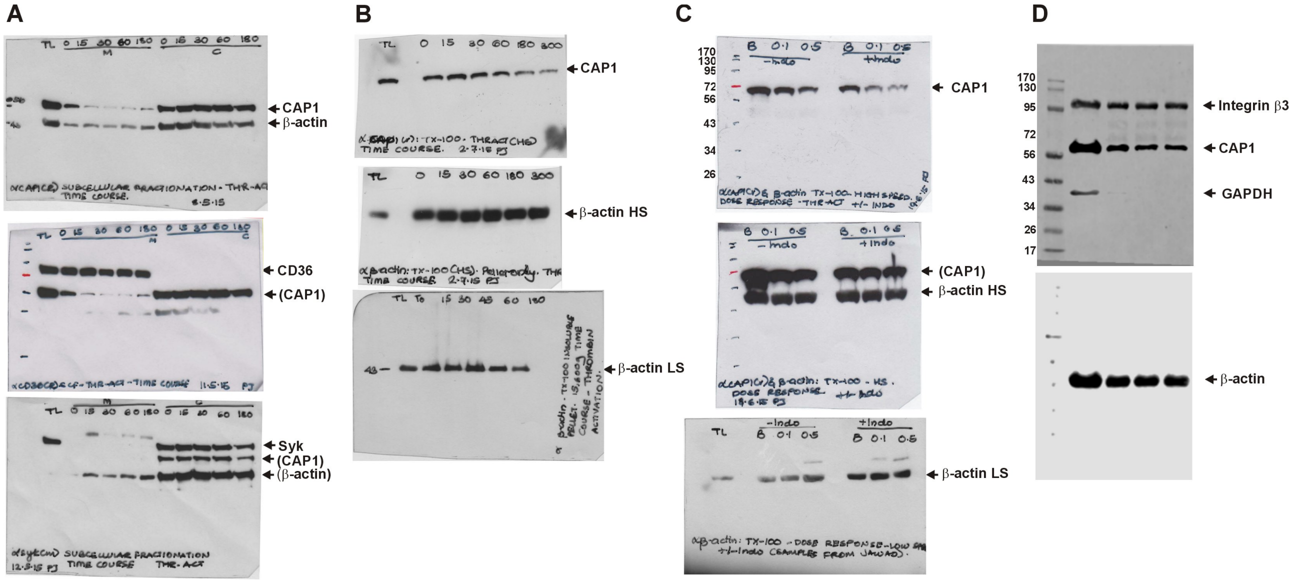

Supplementary Figure S3. Full-length blots corresponding to Figure 4. The blots have been arranged in the same order as they appear in the figure. Note that some blots have been re-probed to visualise further proteins, in which case the previous proteins (in brackets) may appear over or underexposed. The first blot of panels C and D each displays the band sizes of the protein ladder as an example.

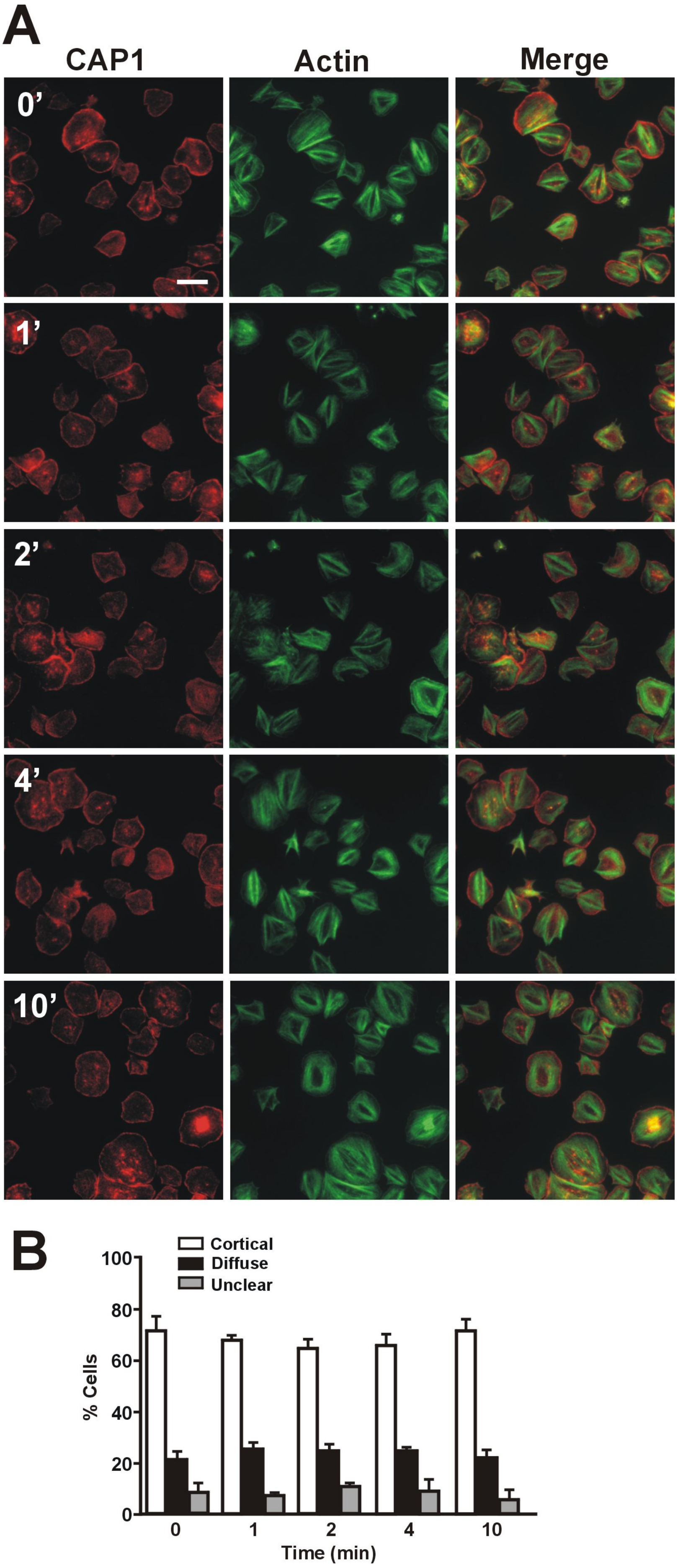

Supplementary Figure 4. Effect of GSK3 inhibition on CAP1 and actin distribution. (A) Platelets were allowed to spread on fibrinogen-coated coverslips and then treated with 2 μM CHIR99021 for the indicated times. Platelets were fixed with paraformaldehyde and immunostained with an anti-CAP1 antibody followed by an Alexa568-coupled secondary antibody (red) and counterstained with FITC-phalloidin for filamentous actin (green). Images were acquired with a fluorescence microscope equipped with a structured illumination attachment. Scale bar 5 μm. (B) Quantification of the pattern of CAP1 distribution. The proportions of cells with predominantly cortical or diffuse distribution of CAP1 in images like the ones shown in A were calculated from 2 to 5 independent experiments each performed in duplicate coverslips. At least 1000 cells per condition in each experiment were scored. Unclear refers to cells that were not sufficiently enough spread to make a judgment. Data are average ± SEM. No significant differences relative to the respective population not stimulated with thrombin, Mann-Whitney test.
